# Somatostatin Analogue Therapy in MEN1-Related Pancreatic Neuroendocrine Tumors from Evidence to Clinical Practice: A Systematic Review

**DOI:** 10.3390/ph14101039

**Published:** 2021-10-12

**Authors:** Anna La Salvia, Franz Sesti, Chiara Grinzato, Rossella Mazzilli, Maria Grazia Tarsitano, Elisa Giannetta, Antongiulio Faggiano

**Affiliations:** 1 Department of Oncology, 12 de Octubre University Hospital, 28045 Madrid, Spain; alasalvi@ucm.es; 2 Department of Experimental Medicine, Sapienza University of Rome, 00161 Rome, Italy; franz.sesti@uniroma1.it (F.S.); grinzato.1774816@studenti.uniroma1.it (C.G.); mariagrazia.tarsitano@uniroma1.it (M.G.T.); elisa.giannetta@uniroma1.it (E.G.); 3 Department of Clinical and Molecular Medicine, Sant’Andrea Hospital, Sapienza University of Rome, 00189 Rome, Italy; rossella.mazzilli@uniroma1.it

**Keywords:** somatostatin analogues, MEN-1, pancreatic neuroendocrine tumors, efficacy, safety, personalized treatment

## Abstract

Neuroendocrine neoplasms (NENs) are relatively rare and complex tumors that can be sporadic or hereditary, as in the context of multiple endocrine neoplasia type 1 (MEN1) where patients display a 70% lifelong risk of developing a pancreatic NENs (pNENs). To date, specific personalized treatment for pNENs in patients with MEN1 are lacking. The aim of this study was to systematically analyze the efficacy and safety of somatostatin analogue (SSA) treatment in patients affected by MEN1-related pNENs. We performed a systematic review of the literature, searching for peer-reviewed articles on SSA (octreotide or lanreotide) treatment in MEN1 associated with pNENs. We selected 20 studies with a pooled population of 105 MEN1 patients with pNENs. Females were 58.5%, median age was 44 years (18–73). TNM stage at diagnosis was stage I–II in 84.8% and stage IV in 15.2%. The overall response rate (SD+PR+CR) was achieved in 88.3% of cases, with stable disease in 75.6% and objective response in 12.7% of patients. The safety profile was favorable with both SSA agents. SSAs appear to be an effective and safe treatment option for MEN1-related pNEN, either at localized or advanced stages.

## 1. Introduction

Neuroendocrine neoplasms (NENs) represent a complex family of tumors arising sporadically or occurring in the context of hereditary syndromes such as multiple endocrine neoplasia type 1 (MEN1), multiple endocrine neoplasia type 2 (MEN2), multiple endocrine neoplasia type 4 (MEN4), Von Hippel–Lindau (VHL) disease neurofibromatosis type 1 (NF1), and tuberous sclerosis [1]. About 10% of NENs are associated with a genetic syndrome [1,2,3,4,5].

Specifically, MEN1 syndrome is characterized by an autosomal dominant transmission, with an incidence of 1:30,000 and a prevalence of 2–3 cases per 100,000 inhabitants, without sex difference. MEN1 syndrome is the result of a germline mutation of the *MEN1* tumor suppressor gene. *MEN1* gene is linked to the chromosomal locus 11q13 [6], is composed of 10 exons, encoding the protein Menin, which plays a key role in cell division, proliferation, and epigenetic regulation [7].

*MEN1* displays a high penetrance with clinical manifestations arising in 80–100% of patients, often with different phenotypes [4]. In the majority of cases, MEN1 manifestations occur before the age of 50, with significant morbidity and reduction of life expectancy [8]. The commonest clinical manifestations of MEN1 include parathyroid adenomas/hyperplasia resulting in primary hyperparathyroidism (in more than 90% of patients), NEN (more frequently within the pancreas, up to 70% of patients), and pituitary adenomas (in about 50% of cases) [9,10].

Considering the complexity of MEN1, a multidisciplinary approach is crucial for an optimal patient management [11]. To date, no preventive treatment is yet available and, therefore, comprehensive surveillance programs aiming at early disease’ detection (by genetic testing) and appropriately timed intervention is mandatory to reduce morbidity and mortality [12].

Unlike sporadic pancreatic NEN (pNENs), MEN1-related pNENs often arise earlier in life; can be multifocal, low-grade (G1 and G2) tumors; and, generally, are characterized by well-differentiated morphology [13]. Similarly to sporadic tumors, pNENs associated with MEN1 can be classified into functioning or non-functioning tumors on the basis of their ability to produce and secrete vasoactive substances into the blood. Among functioning tumors, according to the hormone secreted, different syndromes can be identified. The most relevant are gastrinoma; insulinoma; glucagonoma; or, more rarely, VIPoma syndrome. Gastrinoma is the most commonly identified functioning gastroenteropatic (GEP)-NET, seen in approximately 40% of patients with MEN1, followed by insulinoma at a 10% frequency [10]. Recent data have demonstrated that distant metastases from pNENs are the major cause of MEN1-specific mortality [14]. Additionally, in the context of MEN1, pNENs can occur concomitantly with other MEN1 manifestations, which could contribute to further worsening patient outcomes [15].

Regarding the treatment approach for localized disease in pNENs of patients with MEN1, the international guidelines state that surgery is the only curative option, and a powerful weapon, to alleviate hormonal symptoms in the case of functioning tumors [16]. For patients with locally advanced or metastatic disease, all systemic treatments employed for sporadic tumors can be considered for the MEN1 counterpart [17]. However, due to the lack of specific treatments for pNEN in MEN1 patients, the treatment strategies of these cases are derived from that of sporadic pNENs.

Somatostatin analogues (SSAs), octreotide and lanreotide, represent one of the main systemic treatments for advanced well-differentiated NETs, made manageable thanks to long-acting repeatable (LAR) formulations [18]. SSAs consist of synthetic octapeptides with amino acid sequence analogy to native somatostatin 14 and 28.

SSA indication for NETs has been provided empirically, independently from the anatomical origin, on the basis of their proven activity controlling of hormonal production [19,20], as well as their anti-proliferative activity on the tumor mass [21,22].

Considering other medical treatment for NEN, targeted therapy, including everolimus and sunitinib, has been approved for the treatment of unresectable or metastatic, non-functional G1-G2 NENs in adults with progressive disease [19]. Furthermore, somatostatin receptors (SSTR) may be also targeted with radiolabelled-SSAs such as the β-emitter 177Lu-DOTA-D-Phe-Tyr3-octreotate (177Lu-oxodotreotide or 177Lu-DOTATATE) for peptide receptor radionuclide therapy (PRRT). In this regard, over the past two decades, PRRT has been proven to be an effective and safe therapeutic option in patients with inoperable or metastatic well-differentiated NEN.

Focusing specifically on pNENs, many studies have showed the SSAs’ efficacy in controlling the symptoms related to the hormone secretion of functional tumors, approximately in 50–100% of cases [23,24]. Similarly, several retrospective studies [25,26,27,28,29] and also a phase III randomized clinical trial [22] have clearly demonstrated the activity of SSAs in tumor growth control. The CLARINET study enrolled patients with advanced non-functioning GEP-NENs (except for two patients with gastrinoma in each group of treated and placebo arms), with a Ki-67 index < 10%, and that were indium-111 pentetreotide scan-positive. Progression-free survival (PFS) was significantly increased with lanreotide. The best response achieved with lanreotide was disease stabilization [30]. However, MEN1 patients were not enrolled in the CLARINET study.

The aim of this study was to systematically review the current available data on the efficacy and safety of SSA treatment in patients with pNENs in the setting of MEN1 syndrome.

## 2. Materials and Methods

### 2.1. Search Strategy

**Figure 1:**
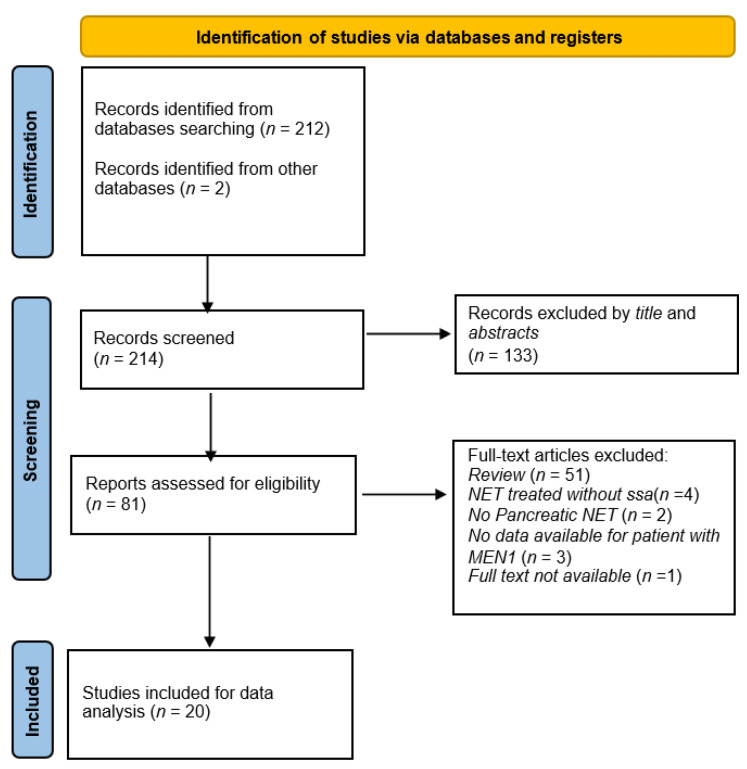
PRISMA flow diagram.

We performed a literature search on electronic databases (PubMed, Science Direct, and Google Scholar) according to the PRISMA Statement 2020 (see Figure 1). The following combinations of keywords relating to the proposed objectives were used: “somatostatin analogues and MEN1”, “SSA and MEN1”, “octreotide and MEN1”, and “lanreotide and MEN1”. Only peer-reviewed research articles (clinical trials and randomized clinical trials) dealing with SSA treatment in MEN1 patients with pNEBs were selected. Original articles in the reference list of included papers were also considered if related to the project aim. No time limit was set.

The following question and the PICO (i.e., Population, Intervention, Comparison, Outcome) elements form the basis that was selected for the review: Are SSA an efficacious and safe option for the treatment of pNENs in MEN1 patients? The question was categorized into PICO elements:

P: MEN1 patients with pNENs;

I: somatostatin analogues (SSAs);

C: alternative to SSA (watch and waiting, other types of interventions such as locoregional treatments, targeted agents, or chemotherapy)

O: effects on tumor growth control.

### 2.2. Selection Criteria

Studies were included according to the following pre-specified criteria:

- Published papers including data regarding efficacy and safety of SSA in patients with pNENs in the context of MEN1syndrome;
- English language;
- Study design: prospective, retrospective, mixed prospective/retrospective trials, and case-report.

### 2.3. Study Selection and Data Extraction

Titles and abstracts of identified articles were independently reviewed by two authors (A.L.S. and C.G.). Irrelevant articles and duplicates were excluded. The full texts for all remaining articles were retrieved. Data concerning study design, study population, clinical characteristics of the included patients (such as age, sex, and concomitant MEN1-related pathological manifestations), type of SSA administered (octreotide or lanreotide), SSA formulation (short-acting, long-acting), SSA schedule of administration, and SSA-related side effects were independently extracted (A.L.S. and C.G.).

## 3. Results

Twenty studies were selected for the systemic revision according with the inclusion criteria. All the studies reported efficacy and safety data of MEN1 patients with pNENs treated with SSAs (octreotide short acting, octreotide LAR or lanreotide LAR). Among the studies included, 55% were case reports (*n* = 11, total subjects = 13) [31,32,33,34,35,36,37,38,39,40,41], 30% were prospective trials (*n* = 6, total subjects = 44) [42,43,44,45,46,47], 10% were retrospective trials (*n* = 2, total subjects = 31) [48,49], and 5% was a prospective/retrospective trial (*n* = 1, total subjects = 17) [50].

**Figure 2:**
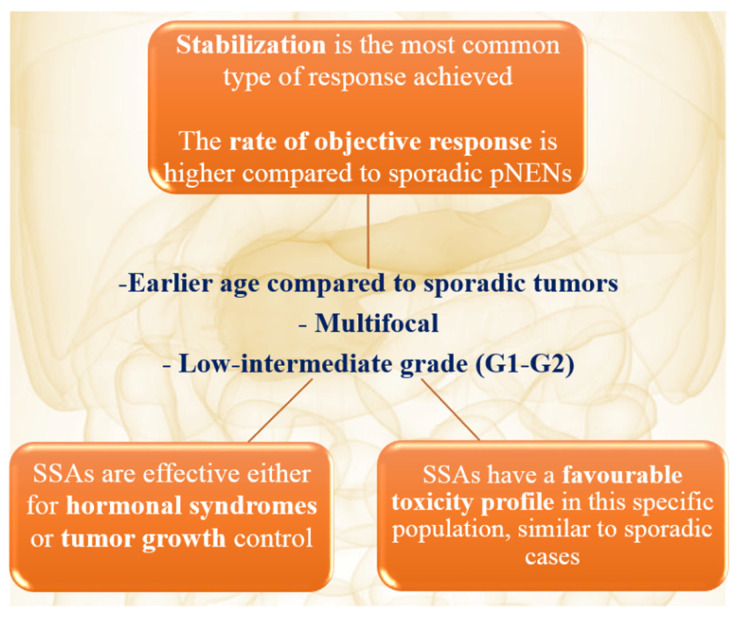
Hot topic: pNENs in MEN1 patients (abbreviations: MEN1 = multiple endocrine neoplasia type 1; pNEN = pancreatic neuroendocrine neoplasm; SSAs = somatostatin analogues).

Overall, the selected studies included a pooled population of 105 patients (median age = 44 years) (18–73) with a diagnosis of one or more well-differentiated pNENs associated with within MEN1 syndrome. The main characteristics of the analyzed patients are shown in Table 1. Sex was reported in 11 articles including a total of 41 cases (women *n* = 24, 58.5%). Thirty-three patients showed at least one other pathological feature related to MEN1, being the association of hyperparathyroidism and pituitary adenoma in the majority of cases (*n* = 17), followed by the hyperparathyroidism alone (15 cases) and pituitary adenoma (1 case). As regards pNENs, TNM staging was available in 92 cases: the majority were diagnosed as stage I tumors (*n* = 73, 79.3%), followed by stage II disease (5 cases) and stage IV (14 cases). The number of pNENs per patient was reported for 64 cases. In 39 of them (61%), the lesions were multifocal. In 54 cases (%), data regarding the surgical removal was reported, with 23/54 undergoing surgical intervention (42.5%). Data regarding tumor functioning was available in 83 cases. Among them, 31 tumors (37.3%) were associated with a hormone secretion and related clinical signs, the most common being the Zollinger–Ellison (in 19/31), followed by glucagonoma (in 6/31), insulinoma (in 2/31), somatostatinoma (in 1 case), and insulinoma associated with gastrinoma (in 1/31); the type of endocrine syndrome was missing in two patients. Therefore, the presenting symptoms were detailed in 12 cases, with fatigue and pyrosis being the two most common clinical manifestations in five and four cases, respectively. The histopathological features were reported in a small percentage of cases. In particular, tumor grade was available in 29 cases, with 10 cases classified as grade 1 and 19 as grade 2 tumors; Ki67 index was available in 9 cases only (Table 1, Figure 2).

Regarding SSA treatment, a different approach has been employed: (i) Short-acting octreotide (50 to 100 mcg/up to three times a day) was administered in 24 cases. (ii) Long-acting release (LAR) octreotide was administered in 73 patients (10 mg every 4 weeks in 8 cases, 20 mg every 4 weeks in 6 cases, and 30 mg every 4 weeks in 38 cases). Octreotide LAR was increased at 30 mg every 3 weeks in two patients and every 2 weeks in two patients. Finally, one patient received octreotide LAR 40 mg every 2 weeks. (iii) Lanreotide autogel in 28 patients, of which 27 at the dose of 120 mg every 4 weeks and 1 at the dose of 120 mg every 8 weeks.

SSA treatment results in stable disease (SD) was found in 59 cases (75.6%), whereas in 10 cases (12.7%), an objective response was achieved: partial response (PR) was described in seven cases (8.9%) and complete response (CR) in three cases (3.8%). The remaining nine cases (11.5%) presented progressive disease (PD). PD was observed in four patients receiving octreotide and five receiving lanreotide. SD was reported in 79.5% of cases treated with octreotide and 64.0% of cases treated with lanreotide. CR was reported in three patients treated with octreotide, whereas no cases were reported with lanreotide.

Adverse effects were reported in 10 cases (9.5%) of the pooled population of patients treated with octreotide or lanreotide. Among them, the majority (*n* = 6, 5.7%) developed gallstones, two presented hyperglycaemia (1.9%), one case reported a gastrointestinal toxicity (nausea and diarrhoea) (0.9%), and one patient reported pain (0.9%). No significant associations were found between side effects and the type of SSA administered. However, the two cases of hyperglycaemia received octreotide as well as the patient who experienced pain, whereas gastrointestinal toxicity was reported in a patient treated with lanreotide.

There was no association between response to SSAs and age (lower/equal than median value vs. higher than median value) (*p* = 0.182), sex (male vs. female) (*p* = 0.651), TNM stage (I-IV) (*p* = 0.103), grade (1–2) (*p*-value = 0.155), Ki67 value (*p*-value = 0.484), or single vs. multifocal tumor (*p* = 0.351). Interestingly, a nearly significant association was found for a better response in functioning tumor (*p* = 0.05): in the 26 cases with functioning tumors, 20 reported SD, 1 reported PR, 3 reported CR, and 2 reported PD.

As regards side effects, no significant associations were found with any of the clinicopathological features.

## 4. Discussion 

To our knowledge, this is the first comprehensive systematic review aimed to investigate the efficacy and safety profile of SSAs in the treatment of pNENs in MEN1 patients.

In 2009, the PROMID study randomized 85 G1 advanced midgut NENs to receive octreotide LAR 30 mg every 4 weeks or placebo [21]. A significant progression-free survival (PFS) improvement was reported for octreotide-treated patients (14.3 vs. 6 months, HR 0.34, *p* = 0.000072) with the following side effects: flushes, diarrhea, flatulence, and abdominal pain.

In fact, on the basis of the results from the CLARINET trial [22] and other retrospective studies, SSAs represent an effective treatment for sporadic pNENs. However, little is known regarding the impact of SSA therapy in patients with pNENs diagnosed in the context of MEN1 syndrome. The aim of this study was therefore to collect all the existing evidence reporting data on the use of SSAs in MEN1-related pNENs.

As a whole, 105 MEN1 patients were found to receive a treatment with SSAs. The overall response rate (SD+PR+CR) was achieved in 88.3% of cases with available data (*n* = 78). Among these, 75.6% were SD, which is similar to the rate reported in sporadic pNEN [22]. Notably, in our analysis, an objective response was achieved in 10 patients (12.7%), with 7 PR and 3 CR. This finding is quite different from those reported in sporadic tumors. In the randomized, placebo-controlled, phase III PROMID trial, enrolling well-differentiated metastatic midgut NENs, 42 treated with octreotide LAR and 43 with placebo, only one patient randomized to octreotide treatment exhibited PR, whereas no complete response occurred [21]. Other studies reported an objective response rate of 4% in 46 patients with sporadic pNENS treated with lanreotide [51]. Similar results (7% PR, no CR) were reported from another phase II study including sporadic GEP NENs [52]. Further evidence obtained with high-dose SSA schedule treatment (defined either as increased dose intensity with lanreotide 180 mg or octreotide LAR 60 mg every 28 days or increased dose density with lanreotide 120 mg or octreotide LAR 30 mg every 14 or 21 days) in 198 NEN patients showed an overall response rate of 84.3%, with 75.7% SD. An objective response was achieved in 8.6% of cases, all presenting PR, while no CR was observed [53]. In addition, in the control arm of the randomized phase III NETTER-1 trial, in which 113 patients with sporadic midgut NEN received octreotide LAR 60 mg every 28 days, a 3% objective response rate (ORR) was reported [54]. Therefore, when the response rate (and in particular the ORR) obtained in sporadic tumors versus MEN1 related NENs are compared, a higher (and perhaps more consistent) benefit for MEN1 patients has to be highlighted. A possible explanation can lie in the high rate of localized disease in the MEN1 pNEN studies, which does not find a counterpart in studies on sporadic tumors [55,56]. Patients with early stage MEN1-related pNENs potentially respond well to “non-aggressive” treatment (such as SSAs) and result in long-term stabilization of tumor growth [3]. On the other hand, SSA therapy in patients with early stage MEN1-related pNENs is well tolerated and does not alter the quality of life (QoL) [48].

**Figure 3:**
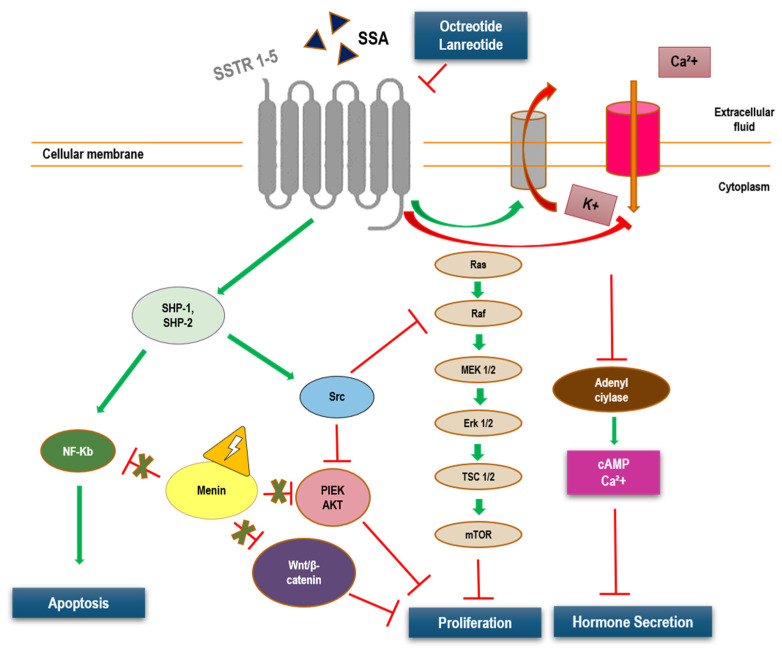
Mechanism of action of SSA in patients with MEN-1. Abbreviations: AKT, protein kinase B; cAMP, cyclic adenosine monophosphate; Erk, extracellular signal-regulated kinase; Ca^2+^, calcium; K+, potassium; MEK, mitogen-activated extracellular signal-regulated kinase; mTOR, mammalian target of rapamycin; PI3K, phosphoinositide 3-kinase; Src, proto-oncogene tyrosine protein kinase Src; SSA, somatostatin analogue; SSTR, somatostatin receptors; TSC, tuberous sclerosis complex.

Second, a biological explanation could be a marked expression of somatostatin receptors (SSTRs) in MEN1-related NENs. In fact, the majority of NENs are characterized by the expression of SSTRs on the cell membrane. Five different G-protein-coupled SSTR subtypes (SSTR 1–5) have been identified to date. The ligand, somatostatin (SST), is a neuropeptide secreted by the GI tract and the brain that regulates neurotransmission, GI motility, hormone secretion, cell proliferation and apoptosis, and immune system modulation (Figure 3).

Among the five SSTRs subtypes (SSTR1–5), SSTR2 and SSTR5 are expressed in approximately 70% pNENs associated with a hormonal syndrome [57]. Octreotide and lanreotide preferentially bind SSTR2, and with less affinity, SSTR3 and SSTR5 [58]. Indeed, Qian et al. found that high expressions of SSTR2 and SSTR3 were more common in tumors with low Ki-67 [59], while absence of SSTR2 correlated with a poor prognosis in pNENs [60]. These data could be applied to MEN1-related pNENs, which are generally low-grade tumors. Thus, stage I-II, low-grade, MEN1-related pNENs are likely to highly express SSTR2. This can be regarded as a positive prognostic factor and, importantly, will retain important predicting values about future response to octreotide and lanreotide treatment, which are known to induce stable disease in most cases, as well as ORR in about 10%.

A clinical benefit on symptoms’ control was also confirmed for MEN1-related functioning pNENs, as in the sporadic counterpart. In MEN1, functioning pNENs seem to be the best candidates to SSAs, as all three patients experiencing a CR had an endocrine syndrome [48,61]. In fact, it is well known that SSAs provide secretory inhibition, symptomatic control, and quality of life improvement [23,62]. However, in the MEN1 setting, the use of SSAs may even prevent the onset of a hormonal syndrome, increasing the long-term favorable effects of these agents on MEN1 patient’ quality of life.

In the present study, no differences in clinical outcomes between different types of SSAs (e.g., octreotide vs. lanreotide) nor relevant associations with clinical and pathological characteristics of the study population were found.

Additionally, the safety profile (for octreotide as well as for lanreotide) appeared similar to that reported for cases of pNENs not associated with MEN1: 78.2% of patients did not report SSA-related side effects and only a minority of subjects showed low-grade and manageable toxicities (with gallstones being the main side effect) [30]. Nor in this case were the observed toxicities associated with the type of SSAs, the schedule of administration, or any of the clinicopathological features considered in this analysis.

Taken together, these data suggest a comparable activity and safety of SSAs for inherited and sporadic pNENs, but a higher rate of tumor shrinkage in MEN1, supporting their wide and early use in these patients.

Some limitations should be cited in interpreting the results of this study: first, the relatively limited sample size (*n* = 105), the higher prevalence of localized rather than metastatic disease, the high percent of missing data for the different variables considered, and the heterogeneity of the treatment schedule with SSAs. Additionally, due to the high number of case reports included (11 of 20 studies), data on important outcomes, such as PFS and overall survival, are lacking. The lack of specific studies on MEN1-related pNENs strongly suggests the usefulness of this review to resume the available data and indicate future direction of clinical research.

## 5. Conclusions

SSAs could be considered an effective and safe treatment option for pNENs in the context of MEN1 syndrome. In this analysis, a higher ORR was observed in MEN1 patients with pNENs treated with SSAs, as compared with to sporadic pNENs. In MEN1, a clear clinical benefit was observed in functioning pNENs, in parallel with the control of tumor growth, which seemed to be higher in patients with an endocrine syndrome. An early use of these compounds in patients with MEN1 is also suggested to prevent further tumor progression and development of endocrine syndrome. No differences in the activity as well as in the toxicity rate have been observed between octreotide and lanreotide, supporting the use of both agents in this population. Additionally, no specific association with clinicopathological features was identified. However, given the limitations of this analysis, prospective studies are highly encouraged to confirm these results, validating the use of SSAs in this specific patient population.

## Figures and Tables

**Table 1:** Patient characteristics.

Characteristic	*n* = 105 (100%)
**Gender**	
Data available	41 (40%)
Men	17 (41.5%)
Women	24 (58.5%)
NA	64 (61.0%)
**Median age**	44 years (18–73)
**Functioning NEN**	
Data available	83 (79%)
Yes	31 (37.3%)
No	52 (62.7%)
NA	22 (21%)
**Type of syndrome in functioning NEN**	
Data available	29 (27.6%)
Gastrinoma	19 (65.6%)
Glucagonoma	6 (20.7%)
Insulinoma	2 (6.9%)
Somatostatinoma	1 (3.4%)
Gastrinoma + insulinoma	1 (3.4%)
NA	76 (72.4%)
**Stage**	
Data available	92 (87.6%)
I	73 (79.3%)
II	5 (5.4%)
III	0 (0.0%)
IV	14 (15.2%)
NA	13 (12.4%)
**Number of pancreatic nodules**	
Data available	64 (60.9%)
1	25 (39.1%)
>1	39 (60.9%)
NA	41 (39.0%)
**Grade**	
Data available	29 (27.6%)
1	10 (34.5%)
2	19 (65.5%)
NA	76 (72.4%)
**Median Ki67 index (%)**	
	2 (1–8)
**MEN1 manifestations**	
Data available	33 (31.5%)
Hyperparathyroidism	15 (45.5%)
Pituitary adenoma	1 (3.0%)
Hyperparathyroidism and pituitary adenoma	17 (51.5%)
NA	72 (68.6%)
**Surgery**	
Data available	54 (51.4%)
Yes	23 (42.6%)
No	31 (57.4%)
NA	51 (48.6%)
**Short-acting SSA therapy**	
Yes	24 (22.9%)
No	81 (77.1%)
**Octreotide LAR**	
Yes	73 (69.5%)
No	32 (30.5%)
**Lanreotide LAR**	
Yes	28 (26.7%)
No	77 (73.3%)
**Response to SSA therapy**	
Data available	78 (74.2%)
SD	59 (75.6%)
PR	7 (9.0%)
CR	3 (3.8%)
PD	9 (11.5%)
NA	27 (25.7%)
**Side effects to SSA therapy**	
No	95 (90.4%)
Gallstones	6 (5.7%)
Hyperglycaemia	2 (1.9%)
Gastrointestinal (nausea, diarrhea)	1 (0.9%)
Pain	1 (0.9%)

Abbreviations: NA = not available.
